# Bilingual Infants Demonstrate Perceptual Flexibility in Phoneme Discrimination but Perceptual Constraint in Face Discrimination

**DOI:** 10.3389/fpsyg.2017.01563

**Published:** 2017-09-12

**Authors:** Leher Singh, Darrell Loh, Naiqi G. Xiao

**Affiliations:** ^1^Department of Psychology, National University of Singapore Singapore, Singapore; ^2^Department of Psychology, Princeton University Princeton, NJ, United States

**Keywords:** bilingualism, phoneme discrimination, face discrimination, perceptual narrowing, other-race effect (ORE)

## Abstract

Perceptual narrowing is a highly significant development associated with the first year of life. It conventionally refers to an orientation toward nativeness whereby infant's perceptual sensitivities begin to align with the phonetic properties of their native environment. Nativeness effects, such as perceptual narrowing, have been observed in several domains, most notably, in face discrimination within other-race faces and speech discrimination of non-native phonemes. Thus, far, nativeness effects in the face and speech perception have been theoretically linked, but have mostly been investigated independently. An important caveat to nativeness effects is that diversifying experiences, such as bilingualism or multiracial exposure, can lead to a reduction or postponement in attunement to the native environment. The present study was designed to investigate whether bilingualism influences nativeness effects in phonetic and face perception. Eleven-month-old monolingual and bilingual infants were tested on their abilities to discriminate native and non-native speech contrasts as well as own-race and other-race face contrasts. While monolingual infants demonstrated nativeness effects in face and speech perception, bilingual infants demonstrated nativeness effects in the face perception but demonstrated flexibility in speech perception. Results support domain-specific effects of bilingual experience on nativeness effects.

## Introduction

One of the most widely documented hallmarks of infant psychological development is perceptual narrowing. Broadly construed, this refers to a nativeness effect whereby infants' perceptual systems attune to environmentally relevant details. At the same time, sensitivity to environmentally irrelevant details often declines. Perceptual narrowing is not specific to a single cognitive domain. Two domains that have been the focus of intensive empirical research on nativeness effects are face perception and speech perception. In both domains, nativeness effects, such as perceptual narrowing, are potentially modified by diversifying experiences. For example, recent research with bilingual infants demonstrates that narrowing may be attenuated by bilingual exposure (Garcia-Sierra et al., 2011; Petitto et al., 2012; Graf-Estes and Hay, 2015; Ferjan-Ramirez et al., 2017). Likewise, diverse experiences with human faces appear to attenuate the course of narrowing in face discrimination (Bar-Haim et al., 2006; Gaither et al., 2012). The current study compares monolingual and bilingual infants in their sensitivity to face and speech contrasts to examine whether bilingual experience modifies perceptual narrowing within *and* across domains. This study bears on a weighty question in infant science (see Scott et al., 2007), specifically, the extent to which perceptual narrowing across domains is driven by a generalized learning mechanism or by domain-specific learning.

Perceptual narrowing in face and speech perception assumes a strikingly similar course of development. In speech perception, there is a broad base of evidence to suggest that infants begin their lives, no matter where they are raised, with universal capacities for speech discrimination (Trehub, 1976; Aslin et al., 1981; Werker et al., 1981; Werker and Tees, 1984; Best et al., 1988; Polka and Werker, 1994). Across a range of language environments, newborn infants exhibit a keen perceptual acuity for differences between speech sounds. However, for many sounds, this ability declines over the first year, most notably for those that are not lexically contrastive in infants' native languages (Werker and Tees, 1983, 1984, but see Kuhl, 2000; Best and Tyler, 2007). There has been an increasing interest in charting the course of development in multilingual children, revealing interesting contrasts with monolingual infants. For example, with respect to vowel perception, bilingual infants appear to undergo a lengthier and more complex pathway to perceptual narrowing (Bosch and Sebastián-Gallés, 2003; Burns et al., 2007; Sundara et al., 2008; Sebastián-Gallés and Bosch, 2009, but see Albareda-Castellot et al., 2011). Further evidence from neurophysiological studies suggests that bilingual infants may retain greater perceptual flexibility than their monolingual peers. More specifically, the time window for perceptual narrowing appears to be both postponed and protracted on account of mastering dual systems (Garcia-Sierra et al., 2011; Petitto et al., 2012; Ferjan-Ramirez et al., 2017). Garcia-Sierra et al. (2011) and Ferjan-Ramirez et al. (2017) demonstrated that bilingual infants begin to respond to native contrasts later than monolingual peers, reflecting a postponement of narrowing. Petitto and colleagues provided neurophysiological evidence via fNIRS that bilingual babies remained open to non-native contrasts for an extended period of time, reflecting a protraction of narrowing (see also Graf-Estes and Hay, 2015; Singh, 2017). This has led to the suggestion that bilingual infants retain a greater openness to phonological contrast in comparison to monolingual peers (Garcia-Sierra et al., 2011; Petitto et al., 2012).

It should be noted that perceptual narrowing is not limited to the perception of vowels and consonants. It is also observed for other types of information, such as lexical tones (Yeung et al., 2014), for signed languages (Baker et al., 2006), for music (Hannon and Trehub, 2005) and relevant to the present study, for face perception. Just as with speech, infants begin with a fine-grained sensitivity to subtle variations in the human face distinguishing both familiar and unfamiliar faces in their environment (Kelly et al., 2007, 2009). Over the next 5–6 months, infants demonstrate a selective decline in face discrimination, notably for faces belonging to relatively unfamiliar racial groups. However, they retain the ability to discriminate faces belonging to individuals of the same race (Anzures et al., 2009). By 9 months, infants therefore start to reliably exhibit the “other-race effect,” whereby other-race faces are no longer discriminated but own-race faces remain discriminable (see also Sangrigoli and De Schonen, 2004; Hayden et al., 2007). As a consequence, over the first year of life, the transition observed for face perception closely resembles that associated with phonetic perception: in both modalities, infants begin with universal sensitivity to contrast followed by experience-dependent decline in sensitivity to contrasts not well represented in their environment.

Analogous to prior investigations of effects of bilingualism on phonetic percpetion, Bar-Haim et al. (2006) explored the effects of racial diversity on face attunement by comparing face discrimination in infants raised in mono-racial environments with those raised in multi-racial environments (the latter were exposed primarily to faces of two races). Infants raised in mono-racial environments developed an own-race face preference by 3 months. However, infants raised in a multi-racial environment did not develop such a preference. This suggests that dual-race face exposure modified the development of a race bias. Consistent with this finding, Gaither et al. (2012) reported that habitual exposure to racial diversity inhibited the development of an other-race effect. Providing an interesting complement to effects of bilingualism on speech perception, these studies suggest that racial diversity may foster greater sensitivity to other-race faces and linguistic diversity may foster greater sensitivity to non-native phonemes. In each domain, diversifying influences appears to reduce perceptual narrowing.

The question remains, however, whether nativeness effects in face and speech perception develop interdependently. There are several similarities in perceptual narrowing for face and speech perception that invite the possibility that both developments may be governed by common mechanisms. For example, both narrowing in face and speech perception transcend medium and modality (i.e., narrowing is observable for non-human faces, music, and signed systems), tentatively suggesting that they may be driven by a domain-general adaptation to experience. Furthermore, there are broad similarities in timing of narrowing in face and speech perception (Scott et al., 2007). In both domains, infants are highly sensitive to face and speech contrasts in the first 6 months of life followed by an environmentally calibrated set of sensitivities between 6 and 12 months (but see Sangrigoli and De Schonen, 2004). Moreover, narrowing in face and speech perception may be regulated by common neural circuitries (see Belin et al., 2011). These similarities in face and speech narrowing have led to the suggestion that these processes are interdependent and may be traced to common evolutionary roots (Pascalis et al., 2014).

In spite of apparent similarities, some have posited different mechanisms underlying narrowing in in face and speech perception. In particular, narrowing in the face appears to be more heavily modulated (and in the event of deprivation, more readily compensated for) by face experience, whereas phonetic perception appears to be more vulnerable to biological constraints and to the timing of exposure (Maurer and Werker, 2014). To date, however, there have been no empirical comparisons of nativeness effects in face and speech perception in infancy. Such an investigation would contribute to our understanding whether perceptual narrowing is driven by a single process or by domain-independent processes.

Given the retentive effects of bilingual exposure on native and non-native linguistic sensitivity, variation in early language exposure potentially provides an opportunity to investigate the interdependence of nativeness effects in face and speech perception. Exposure to multiple languages appears to lead to an attenuation of linguistic narrowing. Likewise, exposure to multiple races appears to lead to an attenuation of face narrowing, but are there cross-over effects? Does bilingualism modify sensitivity to facial contrast as it does linguistic contrast? In an investigation of face sensitivity in bilingual adults, Kandel et al. (2016) explored whether bilingual adults demonstrated the other-race effect widely reported in monolingual adults. Kandel et al. found that bilingual adults did not demonstrate an other-race effect, whereas monolingual adults did. Kandel et al. concluded that infants may demonstrate reduced face narrowing. In this way, early bilingualism may influence the process of perceptual specialization of the developing neurocognitive system, the effects of which are later evident in adulthood.

If face and speech perception varied for bilingual learners, one would expect an attenuation of the other-race effect to be observable in infancy. Moreover, such an account would lead to a hypothesis positing cross-domain attenuation of nativeness effects in both face and speech perception. This question was explored in the current study. Monolingual and bilingual infants were compared on nativeness effects in face and speech perception in a within-subjects design. Infants were tested between 10 and 11 months when perceptual narrowing in face and speech perception has been robustly observed in monolingual populations. It was hypothesized that diversifying effects of language experience arising from bilingual exposure would lead to perceptual flexibility in speech perception (i.e., successful discrimination of native and non-native speech contrasts) as demonstrated in prior research (Garcia-Sierra et al., 2011; Petitto et al., 2012; Graf-Estes and Hay, 2015; Ferjan-Ramirez et al., 2017; Singh, 2017). If nativeness effects in face and speech perception are indeed governed by a single process, it is expected that bilingualism inhibit the other-race effect only in bilingual infants. A single process account of perceptual narrowing would predict that diversifying influences, such as bilingualism, would reduce nativeness effects both in face and speech perception. A domain-specific account of perceptual narrowing would predict effects of bilingualism on speech perception only. This account would predict similar nativeness effects between monolingual and bilingual infants in face perception but not in speech perception.

## Methods

### Participants

Thirty-two 10–11.5 month-old infants were tested for the present study (mean age: 338 days; range: 304–348 days). Sixteen infants were monolingual (at least 90% exposure to English) and sixteen were bilingual (at least 25% exposure to Mandarin Chinese; remaining exposure to English). Parents were interviewed about their infants' language exposure prior to recruitment. This interview consisted of a language exposure questionnaire (Bosch and Sebastián-Gallés, 1997). This questionnaire is a structured interview asking about each person with whom an infant has regular interaction, about the language used by each person, and about the amount of time each person spends with the child. Parents are asked about lifetime exposure and asked about any changes in the language exposure over the child's life. Mean exposure to each language was calculated across all caregivers and for bilingual participants was 51% English (range: 31–75%) and 49% Mandarin Chinese (range: 25–69%. No child had exposure to additional languages. The same calculations were conducted for monolingual infants. Mean exposure to English was calculated across all caregivers and was 95% (range: 90–100%). Some infants had marginal exposure to Mandarin from extended family or day care (average exposure to Mandarin 5%, range: 0–10%). While it would be ideal to have a race matched group with 100% exposure to English, exposure to Mandarin was unavoidable as infants in monolingual group were only included if they were of Chinese race. A criterion of 80–90% exposure to the first language and a minimum of 25% exposure a second language is standard practice to classify infants as monolingual and bilingual respectively in comparison studies in infant speech perception (see Bosch and Sebastián-Gallés, 2003; Sundara et al., 2008; Sundara and Scutellaro, 2011). Such studies often take place in bilingual societies and as such, exclusive exposure to one language is less likely. In light of the goals of the face discrimination data, both groups were carefully controlled for race and for prior race exposure. All infants were of Chinese ethnicity and race. Face exposure was measured via a Facial Contact Questionnaire recording the race of each caregiver over the infants' life. Parents were interviewed about the race of each family member and caregiver with whom the child interacts on a regular basis. All children had exclusive habitual interaction with Chinese faces at home. Five monolingual and five bilingual infants had occasional exposure to other East Asian faces (Malay and Filipino) by non-familial sources (day care provider, domestic helper). Parents were also asked about contact with non-Asiatic faces. Two monolingual infants and two bilingual infants had sporadic contract with Caucasian faces on account of contact with friends. All parents reported no known interaction or exposure to African faces at all. Seven additional infants were tested but excluded from the final data set for incomplete data (6) and technical error (1). This study was carried out in accordance with the recommendations of the National University of Singapore Institutional Review Board with written parental informed consent from all subjects. The study was approved by the National University of Singapore Institutional Review Board. All subjects gave written informed parental consent in accordance with the Declaration of Helsinki.

### Stimuli

#### Speech stimuli

Six tokens of syllables beginning with the voiced bilabial stop /ba/ and the voiced alveolar stop /da/ were recorded as native consonants (contrastive in English and Mandarin) by a female native English speaker. Six tokens each of the Hindi voiceless dental-retroflex (/ta/-/ʈa/) stop consonants were recorded by a female native Hindi speaker. This contrast is not a phonemic distinction in English or Mandarin. These specific contrasts were chosen as they have been widely used to study nativeness effects on speech perception in infancy and have been robustly linked to perceptual narrowing in English monolingual infants (e.g., Werker and Tees, 1984; Werker and Lalonde, 1988; Polka, 1991). Stimuli were produced in infant-directed speech equated for duration, pitch and intensity. Acoustic analyses for speech stimuli are displayed in Table 1.

**Table 1:** Acoustic analyses of auditory stimuli (means and *SD*).

	**Fundamental frequency (Hz) *(SD)***	**Duration (ms) *(SD)***	**Intensity**
Hindi dental stop (ta)	235 (14.8)	711.75 (32.4)	72.5 (0.09)
Hindi retroflex (a)	246 (15.2)	735.92 (43.43)	73.5 (0.08)
English bilabial stop (ba)	251 (13.6)	696.81 (34.31)	72.9 (0.07)
English dental stop (da)	238 (15.1)	748.80 (17.31)	73.1 (0.06)

#### Face stimuli

**Figure 1:**
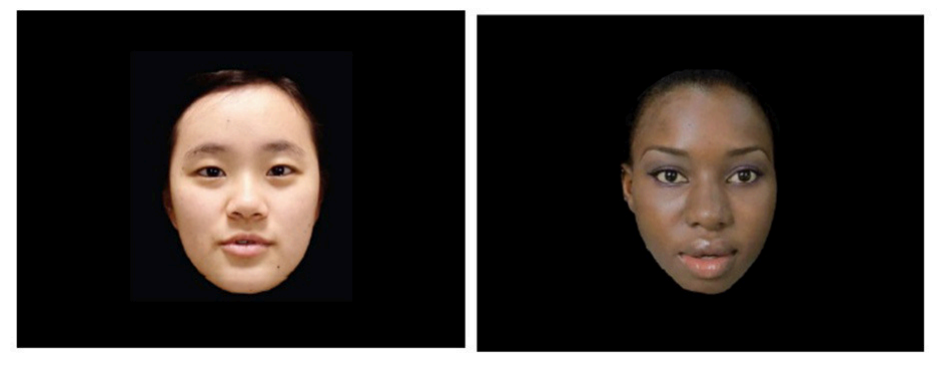
An example of own race faces **(left)** and other-race faces **(right)**.

All faces were female. Four faces were used for each race, two for habituation and one for each test block trial (control and test trial). Faces were cropped to the hairline with the neck obscured and controlled for luminance and brightness (please see Figure 1 for an example of own-(Chinese) and other-(African) race facial stimuli). Attractiveness, representativeness and distinctiveness ratings were collected on each face by 10 Chinese race adults in the country of testing. There were no differences in attractiveness ratings or distinctiveness across faces or between races (*p* = 0.72 for attractiveness, *p* = 0.85 for representativeness, *p* = 0.81 for distinctiveness).

African faces were chosen as the other-race stimulus on account of prior evidence that Chinese infants do discriminate African faces at 3 months, but demonstrate narrowing for African faces, failing to discriminate African faces at 6 and 9 months of age (Kelly et al., 2009). It should be acknowledged that there is prior evidence that African faces may be harder to discriminate for Chinese infants than other ethnic groups, such as Caucasian faces (see Kelly et al., 2009). However, African faces were selected on the grounds that they serve as a closer analog to the Hindi speech contrast: persons of African origin are very rare on the country of testing and like Hindi speech contrasts, African faces were likely unencountered by participants in their native environment. In contrast, Caucasian face exposure is much more probable in the country of testing making effects of prior experience with Caucasian faces on Caucasian face discrimination challenging to control. While there remains the possibility that African faces may have been harder to discriminate than Chinese faces in the present study, the African stimuli used here were provided to us by the authors from past studies showing that these stimuli were discriminated by Chinese infants at 3 months (Kelly et al., 2009) and by Caucasian infants at 3 months (Kelly et al., 2007). We therefore used these stimuli rather than generating our own because these African faces have been shown to be discriminable specifically to Chinese race infants prior to face narrowing. It should be noted that Chinese infants demonstrated earlier narrowing for African faces than for other-race Caucasian faces (Kelly et al., 2009). Likewise, Caucasian infants demonstrated earlier narrowing for African faces than for other-race Chinese faces (Kelly et al., 2009) suggesting that narrowing is race-specific and may occur earlier for African race faces. Further studies could investigate whether bilingual and monolingual infants demonstrate race-specific trajectories for the developmental of the other-race effect. However, based on past studies by Kelly et al. (2007, 2009), we expect that both Chinese and African stimuli used in the present study would be discriminated by both groups earlier in development.

### Procedure

Each infant was administered four habituation tasks (native phoneme discrimination, non-native phoneme discrimination, native (own race) face discrimination, non-native (other race) face discrimination) with task order counterbalanced using a Latin Square rotation. Testing was conducted in a dim, quiet room in an Infant Laboratory. Infants were seated on their parent's lap 70 cm away from a 17” LCD monitor on which the stimuli were presented. The experimenter monitored the session in a control room via closed-circuit television. All parents wore black-out glasses and headphones with masking music for all experiments.

The paradigm was administered using Habit X 1.0 on a Macintosh computer. Each task began with an attention-getter, followed by infant-controlled presentation of the habituation stimulus for each task. In each task, infants were presented with a series of habituation trials, consisting of a syllable (either /ba/ or /da/ for the native discrimination task, or /ʈa/ or /ta/ for the non-native discrimination task). During habituation, multiple tokens of each sound were played drawing from a set of five tokens and cycling through these tokens in random order. The interstimulus interval (ISI) was 750 msec. Habituation stimuli were played continuously in conjunction with a black and white checkerboard until infants looked away (maximum look away time: 1 s, minimum look time: 2 s, maximum look time: 20 s). When the infants' looking time for two consecutive trials declined to a pre-set criterion (50% of the average of the two longest trials), the test phase began. Two test trials were presented in succession: a new token of the habituation stimulus with the visual checkerboard was presented for a fixed length of 10 s (control trial), followed a contrastive stimulus for the same length (test trial) (i.e., a change from /ʈa/ to /ta/ or *vice versa* for the non-native discrimination task and from /ba/ to /da/ or *vice versa* for the native discrimination task). Fixation times to the visual checkerboard for control and test trials were logged. The parameters for each session (habituation criteria, gaze contingent stimulus presentation, sequential test trial presentation) were identical across tasks to ensure parity in task demands. Experimental parameters for the face habituation tasks were exactly the same, except that speech stimuli were absent and face stimuli were presented visually in place of the checkerboard pattern. The experimenter (DL) was blind to condition but was not blind to group. As experimental sessions were not videotaped, trial length was logged on-line and not off-line. This decision was made based on prior studies demonstrating accurate trial length timing using Habit X. However, the present design does not allow for verification of looking times recorded by Habit X.

## Results

**Figure 2:**
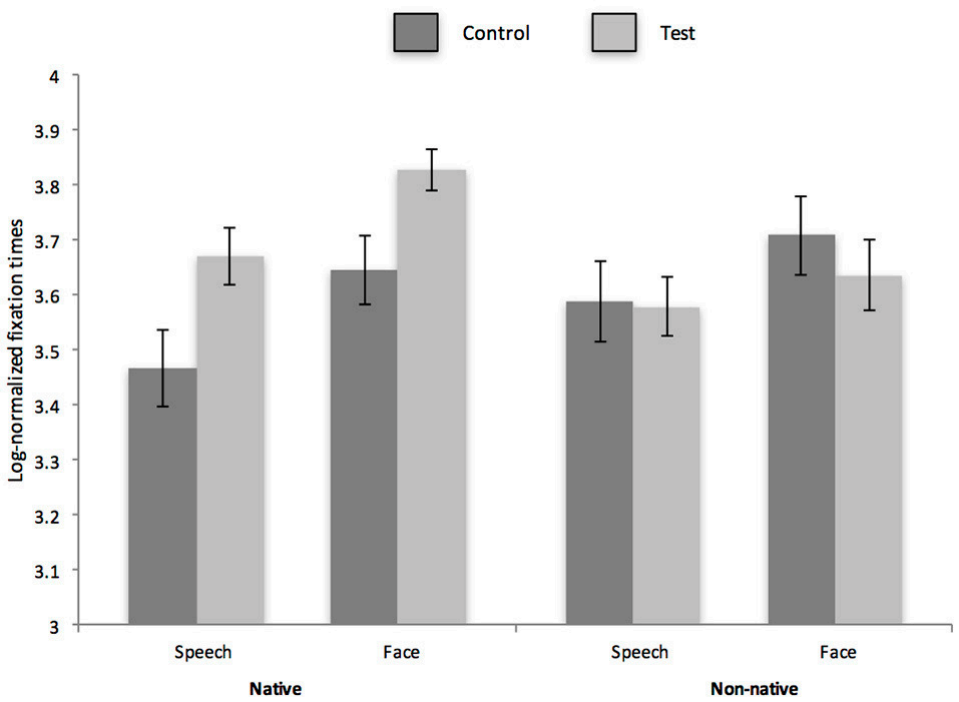
Log normalized fixation times to visual display during control and test trials for native and non-native face and phonetic contrasts for monolingual infants. Error bars reflect S.E.M.

**Figure 3:**
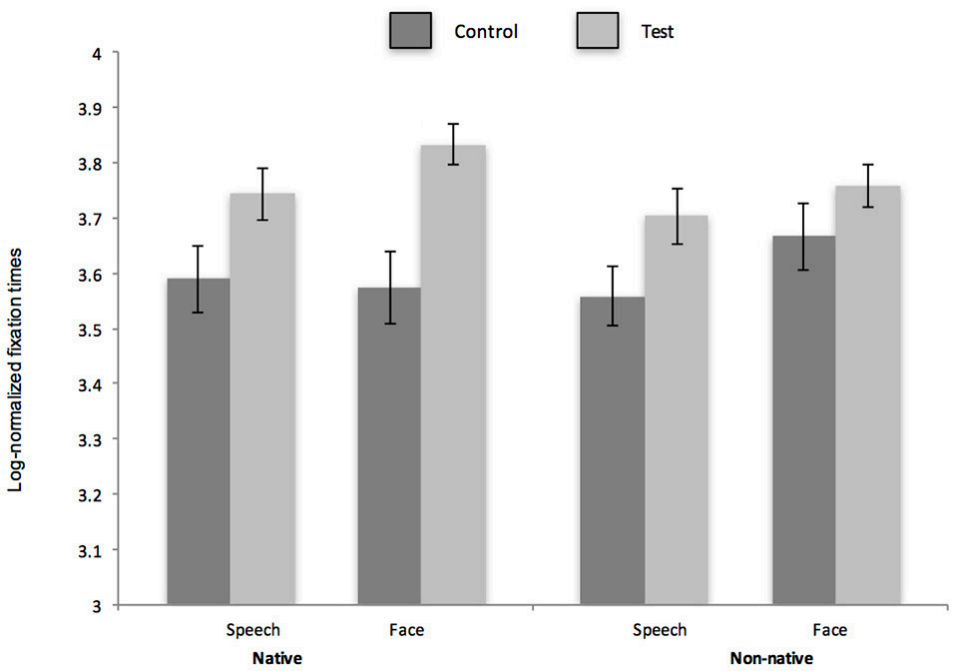
Log normalized fixation times to visual display during control and test trials for native and non-native face and phonetic contrasts for bilingual infants. Error bars reflect S.E.M.

The primary dependent variable was infants' duration of fixation to the checkerboard pattern during each test trial (same and different stimuli) in native and non-native face and speech discrimination tasks. In each task, a significant elevation in fixation to the checkerboard between the first test trial (control trial) and the second test trial (test trial) provides evidence of discrimination. Fixation times are plotted for each task in Figure 2 (monolingual) and Figure 3 (bilingual).

Prior to the primary analysis on fixation times during test trials, a preliminary set of analyses were conducted to ensure that bilingual and monolingual infants demonstrated similar habituation profiles. For each task, monolingual and bilingual infants were compared on their habituation profiles and they did not differ in terms of habituation times, trials to habituation, duration of first habituation trial or duration of last habituation trial (please see Table 2 for habituation data by group and condition and *p* values for comparisons between monolingual and bilingual infants.). Looking times were then compared for control and test trials. As data were not normally distributed, a log transformation was applied to average looking times. A 2 × 2 × 2 × 2 (nativeness: native/non-native stimulus × domain: face stimuli/speech stimuli × test trial type: test/control trial × group: bilingual/monolingual) mixed ANOVA was conducted. There was a significant two way interaction of test trial type and group [*F*_(1, 30)_ = 4.18, *p* = 0.05, partial eta^2^ = 0.12) and a significant two way interaction of test trial type and nativeness [*F*_(1, 30)_ = 12.68, *p* = 0.001, partial eta^2^ = 0.3). Follow up comparisons for the trial type × group interaction revealed that looking times for test trials were significantly higher than control trials for monolingual and bilingual infants [*t*_(15)_ = 2.06, *p* = 0.04, Cohen's d: 0.28) for monolinguals and *t*_(15)_ = 5.3, *p* = 0.00002 for bilinguals, Cohen's d: 0.79]. Moreover, differences in looking time to control and test trials were marginally greater for bilingual infants than monolingual infants [*t*_(15)_ = 1.89, *p* = 0.06, Cohen's d: 0.34]. Follow up comparisons for the trial type × nativeness interaction revealed that looking times for test trials were significantly higher than control trials for native stimuli [*t*_(15)_ = 6.43 *p* < 0.00001, Cohen's d: 0.89) but not for non-native stimuli [*t*_(15)_ = 1.11 *p* = 0.27). Moreover, differences in looking time to control and test trials were significantly greater for native vs. non-native stimuli [*t*_(15)_ = 3.71, *p* = 0.0004, Cohen's d: 0.62). No other effects or higher order interactions were significant. On account of *a priori* evidence of perceptual flexibility in phoneme discrimination in bilingual infants (Garcia-Sierra et al., 2011; Petitto et al., 2012; Graf-Estes and Hay, 2015; Ferjan-Ramirez et al., 2017) and in face discrimination in bilingual adults hypothesized to originate in infancy (Kandel et al., 2016), data from monolingual and bilingual infants were analyzed separately in a series of planned comparisons.

**Table 2:** Mean and *SD* habituation values by group and experiment.

**Group**	**Domain**	**Condition**	**First habituation trial (s)**	**Last habituation trial (s)**	**Number of habituation trials**	**Total habituation time (s)**	**Looking time for control trial (s)**	**Looking time for test trial (s)**
Monolingual infants	Face	Native	11909.19	3694.25	7.88	50564.69	5063.50	7082.31
		SD	5208.96	2066.50	4.98	29295.68	2478.38	2154.28
		Non-native	9531.56	2802.94	6.25	37926.63	5994.94	5056.56
		SD	2928.10	1310.37	2.41	20303.64	2884.70	2712.77
	Speech	Native	12178.69	2627.75	6.38	36950.00	3563.56	5175.38
		SD	5747.41	1061.91	3.12	23268.75	2298.81	2247.26
		Non-native	10077.81	3122.44	6.75	36739.88	4697.00	4249.44
		SD	5133.16	1435.34	3.00	16362.57	2711.18	2090.41
Bilingual infants	Face	Native	9153.69	2715.38	6.50	40338.38	4438.63	7170.06
		SD	4824.23	1145.78	2.97	34748.86	2592.59	2221.92
		Non-native	10944.06	2754.44	6.25	41427.81	5302.19	6093.25
		SD	5404.67	1138.53	2.29	16970.67	2610.15	2150.82
	Speech	Native	12467.88	3749.75	5.13	38165.63	4422.44	6016.56
		SD	5856.49	1793.56	1.26	12400.28	2184.71	2408.05
		Non-native	9778.75	3136.44	7.00	48087.63	4041.00	5487.50
		SD	4890.15	1801.24	2.83	32595.26	1891.28	2066.54
***P*****-values for monolingual/bilingual comparisons from paired samples** ***t*****-tests**.								
Native face			0.14	0.19	0.36	0.42		
Native speech			0.35	0.92	0.98	0.57		
Own race face			0.83	0.06	0.16	0.88		
Other race face			0.88	0.98	0.76	0.25		

### Monolingual infants

A 2 × 2 × 2 (trial type: control/test × domain: face stimuli/speech stimuli × nativeness: native/non-native) repeated measures ANOVA was conducted with fixation times to the screen as the dependent variable. Results revealed a main effect of domain [*F*_(1, 15)_ = 5.1, *p* = 0.04, partial eta^2^ = 0.25) and a significant two-way interaction of nativeness and trial type, *F*_(1, 15)_ = 9.74, *p* = 0.007, partial eta^2^ = 0.39. No other effects or interactions were significant. To investigate our research questions concerning sensitivity to native and non-native face and speech stimuli, a series of planned pairwise comparisons were conducted with a Holm-Bonferroni correction for multiple comparisons. Results revealed a significant increase in fixation time (i.e., successful discrimination) of native race faces and native phonemes {face stimuli: [*t*_(15)_ = 2.89, *p* = 0.01, Cohen's d: 87, speech stimuli: *t*_(15)_ = 3.56, *p* = 0.005, Cohen's d: 8]}. There was no significant increase in fixation time for non-native faces nor for non-native phonemes {face stimuli: [*t*_(15)_ = 1.3, *p* = 0.21, speech stimuli: *t*_(15)_ = 0.13, *p* = 0.9]}.

### Bilingual infants

A 2 × 2 × 2 (trial type: control/test × domain: face stimuli/speech stimuli × nativeness: native/non-native) repeated measures ANOVA was conducted with fixation times to the screen as the dependent variable. Results revealed a main effect of trial type [*F*_(1, 15)_ = 29.75, *p* = 0.0001, partial eta^2^ = 0.67). No other main effects of interactions were significant. To investigate our research question, a series of pairwise comparisons were conducted with a Holm-Bonferroni correction for multiple comparisons. Results revealed a significant increase in fixation time (i.e., successful discrimination) of native race faces and native phonemes {face stimuli: [*t*_(15)_ = 3.9, *p* = 0.001, Cohen's d: 0.61, speech stimuli: *t*_(15)_ = 2.61, *p* = 0.02, Cohen's d: 69]}. Unlike monolingual infants, there was also a significant increase in fixation time for non-native phonemes, [*t*_(15)_ = 2.64, *p* = 0.18, Cohen's d: 67]. Like monolingual infants, there was no significant increase in fixation time between control and test trials for non-native faces [*t*_(15)_ = 1.46, *p* = 0.16]. These findings are consistent with the notion that bilingual infants retain sensitivity to non-native contrasts (Garcia-Sierra et al., 2011; Petitto et al., 2012; Graf-Estes and Hay, 2015; Ferjan-Ramirez et al., 2017) but offers new evidence that they do not retain sensitivity to non-native faces.

Results demonstrate that monolingual and bilingual infants demonstrated a similar capacity to discriminate native speech and face contrasts. However, the groups differed only in their response to non-native speech contrasts: bilingual infants demonstrated sensitivity to a foreign phonetic contrast while monolingual infants did not. With respect to non-native face contrasts, both groups of infants did not discriminate African faces suggesting that both groups demonstrated perceptual narrowing for faces but only monolingual infants demonstrated perceptual narrowing for speech. To confirm group differences with respect to non-native speech discrimination but similarity with respect to non-native face discrimination, looking time differences to control and test trials were compared across groups for Hindi speech discrimination and African face discrimination. Results revealed that bilingual infants demonstrated significantly higher elevation in test trials relative to control trials compared with monolingual peers *t*_(15)_ = 2.5, *p* = 0.03 (Cohen's d: 0.67) in non-native speech perception. In contrast, the groups did not differ in their fixation to test trials relative to control trials for non-native (African) face discrimination, *t*_(15)_ = −0.2, *p* = 0.98.

## Discussion

The present study aimed to investigate face and speech perception in monolingual and bilingual infants. We report three primary findings. First, monolingual and bilingual infants discriminated native faces and native phonemes, demonstrating expected nativeness effects in face and speech perception. Second, monolingual infants demonstrated evidence of perceptual narrowing in speech perception and did not discriminate a non-native Hindi contrast. In contrast, bilingual infants continued to discriminate the same Hindi contrast, demonstrating perceptual flexibility for non-native contrasts. However, perceptual flexibility in speech perception associated with bilingualism did not generalize to face perception: bilingual and monolingual infants demonstrated a comparable other-race effect. Our findings provide preliminary evidence in support of a domain-dependent account of perceptual narrowing based on our findings suggesting that perceptual narrowing in speech and face perception is dissociatively modified by bilingual exposure.

In placing our findings in the context of prior studies, our findings on race sensitivity offer additional insight into those of Kandel et al. (2016) who demonstrated the absence of an other-race effect in bilingual adults. We offer three possible explanations for why an other race effect may be evident in adulthood but not in infancy. First, it is possible that the other race effect develops in monolingual and bilingual learners alike in the early years and that bilingual learners develop increased sensitivity to other-race contrasts as they mature. It is conceivable that substantial bilingual experience is required to “unlock” sensitivity to facial contrast in unfamiliar racial groups. This possibility links to an explanation offered by Kandel et al. as to why bilingual adults may not demonstrate an other-race effect: bilinguals may have to attend to attend more closely to facial cues, such as facial identity, in order to determine which language to use. It is therefore possible that sensitivity to race may emerge from years of experience with using facial information to determine which language to use. Language selection may not be as heavily engaged in preverbal infants and as such, the other-race effect may start to diminish as bilinguals develop a more astute sensitivity to the face as a valuable source of information about language selection. This possibility could be explored by investigating older children's sensitivity to facial variation in own- and other-race groups.

A second possibility is that the other race effect may be attenuated primarily in bilingual groups who encounter greater racial and/or socio-cultural diversity. In the monolingual and bilingual groups sampled by Kandel et al. (2016), nearly half of the bilingual group had parents who spoke a different language and were presumably from immigrant families. In contrast, the monolingual group was relatively homogenous as none of the monolingual sample had traveled for more than 1 month to a foreign country. Moreover, facial contact statistics were not reported, making it hard to determine whether one group had greater experience with the other race used as stimuli (Chinese). Nevertheless, given the statistics that are reported, it is plausible that the bilingual group encountered greater socio-cultural diversity in a way that may have modified their sensitivity to other-race individuals. By contrast, in the present study, all participants and their parents—monolingual and bilingual—were the same race, were citizens, and lifetime residents of the country of testing. The groups therefore did not vary in their national or ethnic origins nor in their residence status. Furthermore, the bilingual group in our sample all spoke English and Mandarin and as such, our sample was more linguistically homogenous than that of Kandel et al. (2016) whose bilingual sample spoke different language pairings. In sum, it is possible that greater socio-cultural and/or linguistic variability in Kandel et al. (2016)'s bilingual sample may have inhibited an other race effect in the bilingual group. This may have been independent of language exposure. A link between high variability and increased perceptual acuity is consistent with a wider literature linking increased variability in the environment—both linguistic and facial—to sharpened perceptual discrimination (Bar-Haim et al., 2006; Singh, 2008; Gaither et al., 2012).

Finally, our study employed African faces as these faces were likely to be entirely unfamiliar to our participants, analogous to the Hindi dental-retroflex phoneme contrast. It is possible that African faces are less familiar to our participants than Chinese faces were to bilingual participants in Kandel et al. (2016). Presumably by adulthood, most adults growing up in Spain and France as in the case of Kandel's participants would not be entirely unacquainted with Chinese race faces, in all likelihood having encountered Chinese faces in person and/or television/movies. By contrast, our participants were indeed entirely unacquainted with African faces as corroborated by parent report. As such, it could be that African faces were much harder to discriminate for Chinese race infants—on account of their total absence in the infants' environment—than Chinese race faces were for European adults. Further comparisons on other race effects using different groups of unfamiliar races may shed light on effects of stimulus-specific factors on the other race effect in bilingual participants.

That bilingual infants retained the ability to discriminate a challenging non-native contrast is consistent with prior studies demonstrating phonological flexibility in bilingual infants in phonetic perception (e.g., Petitto et al., 2012; Graf-Estes and Hay, 2015; Singh, 2017). Indeed bilingual infants have been shown to demonstrate greater flexibility in several cognitive, linguistic and visual domains Kovács and Mehler, 2009a,b; Brito and Barr, 2014). In view of the finding that cognitive flexibility in bilinguals has been reported across multiple domains, it is perhaps surprising that face representations were not rendered more flexible by bilingual exposure. In reconciling domain-general effects of bilingualism on cognitive flexibility reported in the literature (Kovács and Mehler, 2009a,b; Brito and Barr, 2014) with domain-specific influences of bilingualism in the present study, we note that bilingual advantages frequently documented in bilingual children and adults are primarily evident in paradigms where participants learn new information in an experimental setting and then are required to inhibit this very recently learned information (e.g., the Flanker task, Simon task, Stroop task, see (Bialystok, 2009), for a review). We advance the tentative possibility that cognitive flexibility when processing new information acquired *in situ* may be enhanced by bilingual exposure. In contrast, knowledge acquired over the longer term *in vivo* that is the product of perceptual expertise may be relatively resistant to environmental modification, and perhaps even more so to second-degree modification (i.e., modification across domains). The resistance of the products of perceptual expertise to environmental modification may be attributable to the substantial commitment of resources required to build up expertise within a domain, which may fortify these categories against effects of environmental variation. For example, there is evidence that after infants have undergone perceptual narrowing for linguistic contrast, they then require several weeks of repeated exposure to new information to modify learned categories both in speech perception (Kuhl et al., 2003) and in the face (Pascalis et al., 2005). This stands in striking contrast to the few minutes of one-time exposure required to modify newly learned information when acting on new information learned within a laboratory session (e.g., Kovács and Mehler, 2009a,b). It should be noted that this account remains speculative and interactions of domain specificity, extent of expertise, and bilingual exposure merit independent investigation.

In addition to investigating effects of bilingualism on nativeness effects, our study bears on the issue of whether perceptual narrowing across domains is driven by a common mechanism. Our finding that bilingual infants exhibited distinct patterns for face and speech narrowing provides evidence that these processes may develop independently, albeit contemporaneously. If face and speech narrowing were indeed interlocked and unified by a common mechanism, one might expect both domains to be similarly modified by environmental variation. In this way, investigating the effects of diversifying influences in one domain on narrowing in other domains can inform our conclusions about a single- vs. multi-mechanism account of perceptual narrowing. Co-evolution of narrowing for the face and speech, evidenced in the current study, is perhaps expected on the basis of previously attested links between infants' sensitivity to the face and speech (e.g., Tzourio-Mazoyer et al., 2002; Weikum et al., 2013). However, independent pathways to narrowing are consistent with the notion that the course of face and speech narrowing respond in very different ways to stimulus deprivation (Maurer and Werker, 2014), an account of narrowing that is supported by our findings.

The present study sought to investigate cross-domain modification of infant perceptual narrowing by examining effects of bilingual experience on facial and phonetic categories. Results point to stable narrowing in the face in response to bilingual experience and to a modified course of narrowing in the speech on account of bilingual exposure. Dissociative effects of bilingualism on face and speech discrimination provide some support for independent mechanisms governing face and phonetic narrowing. Finally, we suggest that previously reported generalized effects of bilingualism on cognitive flexibility may not extend to knowledge accrued by perceptual expertise.

## Author contributions

All authors designed and conceptualized the study. DL ran the participants through the study and analyzed the data. LS and DL drafted the manuscript.

### Conflict of interest statement

The authors declare that the research was conducted in the absence of any commercial or financial relationships that could be construed as a potential conflict of interest.
